# The Relationship Between Alcohol Hangover Frequency and Hangover Severity

**DOI:** 10.3390/jcm14072428

**Published:** 2025-04-02

**Authors:** Sandra Rîșniță, Emina Išerić, Maureen N. Zijlstra, Ann-Kathrin Stock, Joris C. Verster

**Affiliations:** 1 Division of Pharmacology, Utrecht Institute for Pharmaceutical Sciences (UIPS), Utrecht University, 3584 CG Utrecht, The Netherlands; s.risnita@students.uu.nl (S.R.); e.iseric@students.uu.nl (E.I.); m.n.zijlstra@students.uu.nl (M.N.Z.); 2 Cognitive Neurophysiology, Department of Child and Adolescent Psychiatry, Faculty of Medicine, TU Dresden, D-01307 Dresden, Germany; ann-kathrin.stock@ukdd.de; 3 Centre for Mental Health and Brain Sciences, Swinburne University, Melbourne, VIC 3122, Australia

**Keywords:** alcohol, hangover, severity, frequency, tolerance

## Abstract

**Objective**: Tolerance to the acute effects of alcohol, i.e., feeling less intoxicated after consuming the same amount of alcohol, has been reported for individuals who regularly consume alcohol. In this study, it was investigated whether such tolerance also exists for experiencing the alcohol hangover. **Methods**: Data from five studies that assessed hangover frequency and hangover severity were combined (n = 924). Partial correlations were computed between hangover frequency and hangover severity, with age, sex, and weekly alcohol consumption as possible confounders. **Results**: A significant and positive correlation was found between hangover frequency and hangover severity (r = 0.692, *p* < 0.001). After correcting for sex, age, and weekly alcohol consumption, the partial correlation remained significant (r = 0.526, *p* < 0.001). **Conclusions**: The observed positive association between hangover frequency and hangover severity suggests a reverse tolerance: if hangovers are experienced more frequently, they are more severe.

## 1. Introduction

The alcohol hangover is referred to as the combination of negative mental and physical symptoms which can be experienced after a single episode of alcohol consumption, starting when blood alcohol concentration (BAC) approaches zero [1]. The hangover state is characterized by a plethora of symptoms, including fatigue, headache, and nausea, that can have a negative impact on mood and physical well-being [2] and negatively influence daily activities, such as driving a car [3], riding a bicycle [4], or job performance [5,6,7].

The economic costs of hangovers for the Dutch economy in terms of absenteeism (i.e., days not worked due to hangover) and presenteeism (i.e., being 24.9% less productive on average on hangover days) were estimated at EUR 2.7 billion for 2019 [7]. In addition to economic costs, the alcohol hangover is a serious public health concern. Frequently experiencing hangovers makes individuals more susceptible to developing depression [8,9], ischemic stroke [10], and cardiovascular disease [11]. In addition, it has been hypothesized that frequently experiencing hangovers predisposes individuals to developing alcohol use disorder in the future [12,13,14].

It remains an unanswered question whether the relationship between hangover frequency and hangover severity is positive or negative. That is, do hangovers become worse when you experience them more frequently (i.e., reverse tolerance), or do they become less severe because you get used to the amount of alcohol consumed (i.e., tolerance)?

Schuckit and colleagues published extensively on the development of tolerance to acute alcohol effects. Drinkers who start to score lower on the Self-Rating of the Effects of Alcohol (SRE) Scale need to consume more alcohol to reach the same intoxication effect as they previously experienced [15,16,17,18,19]. Extrapolating the concept of tolerance seen for acute alcohol effects to the alcohol hangover, it could be hypothesized that when experiencing hangovers more frequently, after consuming the same amount of alcohol, these hangovers would become less severe (i.e., tolerance), thereby potentially fostering a further escalation of alcohol consumption.

Alternatively, reverse tolerance could develop when hangovers are experienced more frequently. Išerić et al. [20] recently discussed the hypothesis that with more frequent hangovers, the chances of developing chronic systemic inflammation increase. Systemic inflammation has been linked to poorer health and increased susceptibility to developing chronic diseases, such as diabetes and cardiovascular disease. This condition may also increase the likelihood of experiencing hangovers, and due to the reduced immune fitness, the severity of these hangovers may increase. As a result, a positive association between hangover frequency and hangover severity would be expected: more severe hangovers when they are experienced more frequently.

Only a few studies have evaluated the relationship between hangover frequency and hangover severity. In an experimental study in 90 social drinkers, Köchling et al. [21] found no significant correlation between hangover frequency and reported hangover severity after administering alcohol to reach a BAC of 0.12%. However, for the correlation between hangover frequency and hangover severity, this study used a categorical measure of hangover frequency (‘rarely’, ‘once monthly’, ‘more than once monthly, and less than once weekly’, ‘once weekly’, and ‘more than once weekly’) and an unvalidated composite symptom severity score. The latter may explain the absence of a significant correlation. Verster et al. [22] summarized assessments of three studies relating hangover frequency to hangover severity. The first survey study among 791 Dutch students [23] related hangover frequency to the severity of the past month’s most recent hangover occasion. Hangover severity was assessed via three different validated composite hangover symptom severity scales, the Hangover Severity Scale [24], the Acute Hangover Scale [25], and the Hangover Symptom Severity Scale [23]. A significant positive correlation was found for each severity scale (r = 0.145 to r = 0.198, *p* < 0.001), which remained significant after correcting for the amount of alcohol consumed. The second survey among 333 international young adults on holiday or working in Fiji [26] related hangover frequency to the past three days’ average hangover severity. A significant positive partial correlation, corrected for the estimated BAC, was found between hangover frequency and hangover severity (r = 0.276, *p* < 0.001). The third study was a naturalistic study [27], in which 99 Dutch students consumed alcohol freely at a venue of choice. The next day hangover severity was assessed with the HSS and a single-item rating scale, ranging from absent (score 0) to extreme (score 10) [28]. Again, significant correlations were found between the hangover frequency and hangover severity, assessed with the Hangover Severity Scale (r = 0.452, *p* < 0.001) and the single-item hangover scale (r = 0.529, *p* < 0.001). The correlations remained significant after correcting for the estimated BAC (HSS: r = 0.301, *p* = 0.004, single-item: r = 0.297, *p* = 0.004).

Except for Köchling et al. [21], all other studies reported a significant positive correlation between hangover frequency and hangover severity. There are, however, some limitations to these studies. First, all studies except the research conducted in Fiji [26] assessed hangover severity for a single drinking occasion. The latter is a disadvantage of the study methodology, as a single drinking occasion does not necessarily reflect the average hangover severity these individuals experience and is therefore not necessarily representative. For the individual drinker, hangover severity can vary between drinking occasions. For example, Köchling et al. [21] reported that there was a significant within-subject variation in hangover severity in their study: about 20% of the sample reported very different hangover severity ratings on each test day, despite the fact that an equal amount alcohol was consumed on the assessed occasions [29]. Therefore, it is more accurate to access an average hangover severity score corresponding to multiple hangover occasions, than assessing hangover severity at a random single hangover occasion. Secondly, all studies except for Fuit et al. [27] assessed hangover severity with different composite symptom scales. As each of these scales include different symptoms, it is unlikely that the sum score accurately reflects the overall hangover severity [28]. To determine the relationship between hangover frequency and hangover severity more accurately, we combined data from five studies that assessed both hangover frequency and the average hangover severity, all using the preferrable single-item hangover severity scale [28]. The objective of the current study was to evaluate the relationship between hangover frequency and average hangover severity.

## 2. Materials and Methods

A literature search (PubMed and cross references) was conducted with the key words ‘hangover frequency’ and ‘hangover severity’ to identify studies that assessed both hangover frequency and average hangover severity. There were no other inclusion or exclusion criteria. The search retrieved n = 40 publications. N = 35 publications were excluded as they did not include assessments of both hangover frequency and average hangover severity. Data from five studies were included and combined into one dataset [30,31,32,33,34]. The study characteristics are summarized in Table 1. The first study comprised a survey among 557 Dutch students [30]. The second study comprised a survey among 341 Dutch students, PhD students and post-docs [31]. The third study comprised a survey among 108 Dutch students [32]. The fourth study was a survey among 317 young adults living in Germany [33]. The fifth study comprised a study among 161 Dutch students [34].

All studies assessed hangover frequency by asking ‘How many hangovers did you experience per month?’ and the corresponding hangover severity was assessed with a single-item scale, ranging from 0 (absent) to 10 (extreme) [28]. Age (years), sex (male or female), and average weekly alcohol consumption in standard units were also recorded.

The statistical analysis was performed with SPSS (IBM Corp. Released 2013. IBM SPSS Statistics for Windows, Version 29.0. IBM Corp., Armonk, NY, USA). Mean and standard deviation were computed for each variable. Normality was tested with the Shapiro–Wilk test. The hangover severity and frequency data are not normally distributed (*p* < 0.001). Therefore, nonparametric statistical tests were used to analyze the data. Sex differences were evaluated with the Independent Samples Mann–Whitney U Test. Differences between males and females were considered statistically significant if *p* < 0.05.

Spearman’s correlation was computed between hangover frequency and hangover severity. In addition, a nonparametric Spearman’s partial correlation [35,36] correcting for age, sex, and weekly alcohol consumption was computed. Finally, nonparametric Spearman’s partial correlations between age and hangover frequency as well as severity were computed, correcting for the amount of weekly alcohol consumption. Correlations were considered statistically significant if *p* < 0.05. The analyses were conducted for the combined sample and for males and females separately.

## 3. Results

Of the total n = 1484 participants, n = 924 reported to consume alcohol and provided data on their hangover frequency and average severity. Their mean (SD) age was 21.8 (3.6) years old, and 27.9% of the samples were male. They consumed on average 7.6 (9.0) alcoholic drinks per week, and reported 1.4 (2.1) hangovers per month, with an average hangover severity rating of 3.2 (2.7). A comparison of these outcome variables between males and females is summarized in Table 2. Males consumed significantly more alcohol per week and reported significantly more hangovers. No significant sex difference was found for hangover severity, and although statistically significant, the age difference between the sexes was neglectable (i.e., less than 2 months on average).

**Figure 1:**
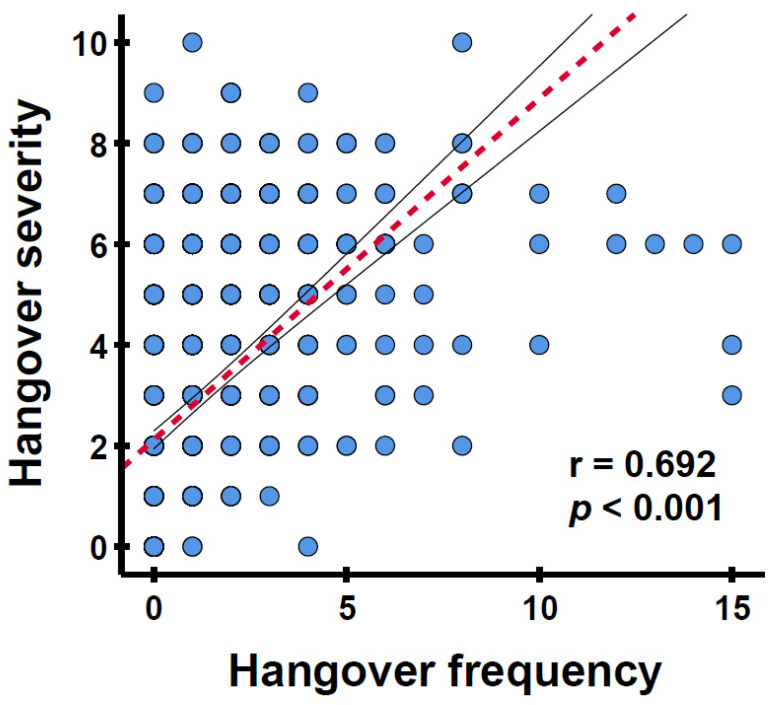
The relationship between hangover frequency and average hangover severity. Hangover frequency was assessed as occasions per month; hangover severity was rated on a scale ranging from 0 (absent) to 10 (extreme). The red striped line represents Spearman’s correlation. The black lines indicate the 95% confidence interval.

For the individual studies, all correlations between hangover frequency and hangover severity were statistically significant (Refs. [30, 31, 32, 33, 34]: r = 0.624, r = 0.824, r = 0.828, r = 0.853, and r = 0.850, respectively, all *p* < 0.001). For the combined sample, the correlation between hangover frequency and hangover severity, as depicted in Figure 1, was positive and significant (r = 0.692, *p* < 0.001). After correcting for sex, age, and weekly alcohol consumption, the partial correlation remained significant (r = 0.526, *p* < 0.001). The correlation between hangover frequency and hangover severity was significant for both males (n = 269, r = 0.729, *p* < 0.001) and females (n = 681, r = 0.674, *p* < 0.001) and remained significant after correcting for age and weekly alcohol consumption (r = 0.617, *p* < 0.001 and r = 0.487, *p* < 0.001, respectively).

Finally, nonparametric partial correlations, correcting for weekly alcohol consumption, were computed between age and hangover frequency and hangover severity. The correlations between age and hangover frequency (r = 0.071, *p* = 0.029) and between age and hangover severity (r = −0.002, *p* = 0.946) were not statistically significant. For males and females separately, the correlations of age with hangover frequency and hangover severity did not reach statistical significance.

## 4. Discussion

The analyses showed a significant and positive correlation between hangover frequency and hangover severity. This finding was observed for both the individual studies and the combined sample, for males and females separately, and remained significant after correcting for age, sex, and weekly alcohol consumption. This consistent observation of a positive association between hangover frequency and hangover severity suggests the development of a reverse tolerance, i.e., when hangovers are experienced more frequently, they tend to be worse.

The observation of a reverse tolerance is opposite to the effects seen for the acute effects of alcohol, where tolerance may develop when alcohol is consumed more frequently [15,16,17,18,19]. The findings are in line with the hypothesis of Išerić et al. [20], that the development of systemic inflammation and the corresponding reduced immune fitness may exaggerate hangover severity, as the hangover is elicited by an inflammatory response to alcohol consumption [37] that may be greater in individuals who (already) have elevated immune biomarker levels due to frequently drinking alcohol and/or experiencing hangovers [20]. Future prospective studies assessing biomarkers of immune functioning should confirm this hypothesis.

Strengths of this study comprise its large, combined sample size and the fact that we corrected the correlation between hangover frequency and hangover severity for age, sex, and weekly alcohol consumption. The latter is important, as previous research has shown that on average both alcohol consumption as well as hangover frequency and severity decrease when growing older [38,39,40]. In addition, sex differences have been reported, showing a greater hangover frequency and severity in males compared to females [40]. However, these sex differences may be caused by the greater alcohol intake of males compared to females, and other research suggested that sex differences are no longer significant after correcting for alcohol intake [41]. Indeed, research showed that for both males and females, hangover frequency and severity increase with greater alcohol intake [42]. A second strength of this study was the use of a single-item overall assessment of hangover severity, which is considered more accurate than composite symptom scores [28].

A limitation of this study includes the fact that there are several other factors that might influence overall alcohol consumption and, in particular, hangover frequency and/or hangover severity that were not taken into account, since these were not (consistently) measured across the studies. These include, but are not limited to, lifestyle factors such as sleep [43], daily diet [44], physical activity [45], smoking and drug use [27,46], congener content of the consumed drinks [43,47], estimated BAC [26], subjective intoxication while drinking [26,48,49], mood while drinking [26], race and ethnicity [50,51], genetic predisposition [50,52,53], and familial risk for alcoholism [54,55,56,57]. Future research should take these factors into account. A second limitation of the current work is the fact that all survey data were collected retrospectively. This could have introduced recall bias among participants. It is therefore important that future prospective, longitudinal studies including real-time momentary assessments confirm our findings. It may also be interesting to further investigate other characteristics of the hangover episode, such as its duration related to the global single-item hangover severity scores. Third, data from different studies were combined for the presented analysis. This may introduce heterogeneity, as the studies were conducted in different countries, used different recruitment strategies, and included different age groups and participants (e.g., students versus the general adult population). This was however performed on purpose, as it was aimed at creating a diverse sample. Of importance, the assessments of hangover frequency and hangover severity were identical across all studies. Fourth, all studies were cross-sectional, and correlational analyses were conducted. In theory, correlations can be interpreted bi-directionally. However, as it is unlikely that experiencing more severe hangovers will lead to having hangovers more frequently, we interpret the study outcome that more frequently experiencing hangovers is associated with having more severe hangovers. Fifth, data from five individual studies were combined into one dataset. Therefore, there is a potential risk that heterogeneity between the studies has influenced the overall outcome. However, there are several factors that reduce or eliminate this impact. Of importance in this context is the fact that each study assessed hangover frequency and severity in the same way. Further, the correlations between hangover frequency and hangover severity of the individual studies were highly comparable with each other and the overall analysis. Sixth, while the study outcomes are clear, the correlational analyses do not provide biological evidence for the causes of the observed relationship between hangover frequency and hangover severity. Išerić et al. [20] suggested that with more frequent hangovers, the chances of developing chronic systemic inflammation increase. To prove this hypothesis, future longitudinal studies should be conducted, including the assessment of biomarkers of immune functioning. A final limitation of the current sample is the age range from 17 to 44 years. Although this is the age range at which hangovers are most frequently experienced [39], future research should also investigate the alcohol hangover in older age groups.

## 5. Conclusions

Notwithstanding the limitations discussed above, the data show a significant and strong positive correlation between hangover frequency and hangover severity. The observation of a reverse tolerance deserves more attention by researchers and policymakers, as frequently experiencing hangovers has a significant impact on the susceptibility to develop chronic systemic inflammation and related immune-related diseases.

## Figures and Tables

**Table 1:** Sample characteristics.

Reference	Combined	[30]	[31]	[32]	[33]	[34]
Country	The Netherlands, Germany	The Netherlands	The Netherlands	The Netherlands	Germany	The Netherlands
Data collection	2014–2023	2014	2021	2021	2021–2022	2023
Recruitment method	E-mail, face-to-face	Face-to-face	Email	Face-to-face	Email, face-to-face	Email
Survey format	Paper, online	Paper	Online	Paper	Online	Online
Population	Students and young adults	Students	PhDs, students, and postdocs	Students	Young adults	Students
N	1484	557	341	108	317	161
Male/female (%)	27.6/72.4	28.1/71.9	24.9/75.1	28.7/71.3	32.5/67.5	21.9/78.1
Mean (SD) age	22.4 (3.9)	20.8 (2.6)	23.0 (4.2)	21.5 (2.6)	25.5 (4.1)	20.6 (1.8)
Age range (years)	17–44	18–30	18–44	17–30	18–35	18–26
Mean (SD) alcohol units per week	7.1 (8.8)	7.4 (8.7)	6.4 (8.3)	9.6 (8.8)	6.3 (10.5)	5.3 (5.2)
Mean (SD) hangover frequency	1.3 (2.1)	1.1 (1.9)	1.5 (2.2)	2.5 (3.1)	1.2 (1.7)	1.3 (1.6)
Mean (SD) hangover severity	3.0 (2.7)	3.4 (2.8)	2.7 (2.7)	3.1 (2.7)	2.2 (2.6)	2.7 (2.4)

**Note:** Hangover frequency was assessed as occasions per month; hangover severity was rated on a scale ranging from 0 (absent) to 10 (extreme).

**Table 2:** Study outcomes.

	Overall	Males	Females	*p*-Value
Number of participants	924	255	660	
Age (years)	21.8 (3.6)	22.5 (4.0)	21.6 (3.4)	0.001 *
Mean (SD) alcohol standard units per week	7.6 (9.0)	12.0 (12.8)	5.8 (6.1)	<0.001 *
Mean (SD) hangover frequency	1.4 (2.1)	1.9 (2.5)	1.2 (1.9)	<0.001 *
Mean (SD) hangover severity	3.2 (2.7)	3.5 (2.8)	3.1 (2.7)	0.058

Hangover frequency was assessed as occasions per month; hangover severity was rated on a scale ranging from 0 (absent) to 10 (extreme). Nine participants did not report their sex but were included in the overall sample. Sex differences were considered statistically significant if *p* < 0.05, indicated by *.

## Data Availability

The data are available from the corresponding author upon reasonable request.

## Abbreviations

The following abbreviations are used in this manuscript:

BAC	Blood alcohol concentration
SRE	Self-rating of the effects of alcohol
